# Experience with 6-month paliperidone palmitate in a mental health center: descriptive study in real clinical practice

**DOI:** 10.1192/j.eurpsy.2024.1523

**Published:** 2024-08-27

**Authors:** I. Garcia Del Castillo, A. P. Balaguer, B. G. Esteban, M. V.-B. García del Castillo, H. V.-H. García del Castillo, S. Castelao-Almodovar, A. Arce de la Riva, F. Neira Serrano

**Affiliations:** ^1^Psychiatry, El Escorial Hospital, El Escorial; ^2^Psychiatry, Puerta de Hierro University Hospital; ^3^Statistics, Francisco de Vitoria University, Madrid, Spain

## Abstract

**Introduction:**

Extensive evidence supports that the use of long-acting inyectable antipsychotics (LAIs) reduces the risk of relapses and maintains functional and symptomatic improvements in patients with schizophrenia, both in the initial stages and in chronic cases. Several LAIs are available but paliperidone palmitate is the only antipsychotic with formulations lasting 3 (PP3M) and 6 (PP6M) months. Longer-duration LAIs achieve stable treatment with fewer injections. Recent studies with PP3M support a reduction in hospitalizations and emergency room visits compared to monthly paliperidone and aripiprazole or oral antipsychotics.

PP6M seems to be at least as effective and well tolerated as other LAIs in preventing relapses in previously stabilized patients with schizophrenia.

**Objectives:**

to assess efficacy and tolerability of PP6M in a real clinical practice compared to previous treatment (oral antipsychotics or other LAIs)

**Methods:**

Patients with a diagnosis of psychotic disorder and treatment with PP6M have been recruited consecutively in a Mental Health Centre in the Community of Madrid (Spain). Clinical stability (CGI and emergency visits and hospitalizations since the start of treatment), tolerability (adverse effects), functionality (PSP scale) and satisfaction with treatment (TMSQ scale) have been studied.

**Results:**

16 patients were included throughout the first 6 months of treatment with PP6M treated at a CSM in the Community (CSM) of Madrid, of which 2 abandoned the study. Among the 14 patients included, aged between 26 and 60 years, 13 had a diagnosis of schizophrenia and one of schizoaffective disorder (according to DSM5). No significant adverse effects were recorded, except for pain at the injection site. The majority were psychopathologically stable patients - 2 of them of recent onset (up to 36 months of evolution) and 7 psychopathological decompensations, measured as visits to the emergency room or psychiatric readmissions, have been detected during the first 6 months of follow-up in CSM. All patients had previously been admitted to treatment with PP6M (minimum 1 admission, maximum 20 admissions). The results of the baseline scores obtained on the psychometric scales applied were: CGI (15.35/35), PSP (62.78/100) and TMSQ (53.35/80).

**Conclusions:**

The existing scientific evidence to date indicates that the application of PP6M is giving safe results in the first months of follow-up, with few side effects recorded, and a low rate of decompensations. This study based on data from real clinical practice in a CSM, despite the limitation due to the small sample size, obtains similar results consistent with those described in previous clinical trials.

**Disclosure of Interest:**

I. Garcia Del Castillo Paid Instructor of: Training on drug use paid, A. Balaguer: None Declared, B. Esteban: None Declared, M. García del Castillo: None Declared, H. García del Castillo: None Declared, S. Castelao-Almodovar: None Declared, A. Arce de la Riva: None Declared, F. Neira Serrano: None Declared

